# The role of women's empowerment in the uptake of maternal health services in low- and middle-income countries: a propensity score-matched analysis

**DOI:** 10.7189/jogh.15.04188

**Published:** 2025-06-20

**Authors:** Daniel Gashaneh Belay, Gizachew A Tessema, Jennifer Dunne, Aditi Roy, Richard Norman

**Affiliations:** 1 Curtin School of Population Health, Curtin University, Perth, Western Australia, Australia; 2 Department of Epidemiology and Biostatistics, Institute of Public Health, College of Medicine and Health Sciences, University of Gondar, Gondar, Ethiopia; 3 School of Public Health, University of Adelaide, Adelaide, South Australia, Australia; 4 Dementia Centre of Excellence, enAble Institute, Curtin University, Perth, Western Australia, Australia

## Abstract

**Background:**

Women’s empowerment directly influences the quality and timeliness of the maternal health care they receive; a lack thereof, particularly in low- and middle-income countries (LMICs), is likely to contribute to poor uptake of maternal healthcare. We aimed to evaluate the role of women's empowerment in maternal healthcare in LMICs.

**Methods:**

We used the recent Demographic and Health Survey (DHS) data on 71 077 married/partnered women from 35 LMICs. We categorised women as empowered if they participated in all decision-making activities and were able to disagree that a husband is justified in hitting or beating his wife for any reason. We used logit propensity score matching (PSM) analysis to estimate the effect of women's empowerment on maternal health services.

**Result:**

Only one-third (33.8%) of reproductive-age women in LMICs (95% confidence interval = 27.7–40.8) were estimated to be empowered. Women’s empowerment was associated with an 11.2 percentage point increase in having adequate antenatal care (ANC) visits (average treatment effects on the treated (ATT) = 0.112, standard error (SE) = 0.026) and an 8.0 percentage point increase in the likelihood of health facility childbirth (ATT = 0.078, SE = 0.039). However, there was insufficient evidence for early postnatal care visits.

**Conclusions:**

Empowering women has a positive association with the utilisation of adequate ANC visits and health facility childbirth in LMICs. These findings underscore the necessity for public health programmes to empower women and enhance their decision-making abilities to improve maternal healthcare uptake, such as health facility childbirth and ANC visits.

Women’s empowerment is a global issue and can be defined as the fostering of a woman’s sense of self-worth, decision-making power, and access to opportunities and resources [1]. Empowering women is the process of enhancing their capacity to make purposeful choices and transform them into desired actions and outcomes [2,3]. However, in regions where women’s empowerment and gender equity pose challenges, the decision-making ability of women is limited, contributing to poor uptake of maternal health services [2,4]. Thus, empowering women and communities is one of the guiding principles of the World Health Organisation (WHO) for ending preventable maternal mortality in low- and lower-middle-income countries [5].

Low- and middle-income countries (LMICs) accounted for 95% of the global preventable maternal mortality related to pregnancy and childbirth complications in 2020 [6]. In the same year, the maternal mortality ratio in low-income countries was 430 per 100 000 live births compared to 13 per 100 000 live births in high-income countries [6]. These deaths can be prevented or minimised by providing high-quality care throughout pregnancy, childbirth, and the postpartum period [7]. Maternal healthcare uptake was found to be insufficient in LMICs, with only 68% of births in low-income and 78% in middle-income countries assisted by skilled health personnel [8]. Several influencing factors, such as geographic, economic, and cultural beliefs [9], as well as low levels of women's empowerment [10], could affect the overall utilisation of maternal healthcare facilities.

The associations between women’s empowerment and some aspects of their health, such as fertility [11] and contraception preference [12,13], have been previously investigated. While some evidence links women's empowerment to maternal healthcare uptake [14,15], existing studies have primarily focussed on individual components of maternal healthcare rather than comparing the effect of women's empowerment on the three maternal health services (antenatal care (ANC), health facility delivery, and postnatal care (PNC)) simultaneously. This approach helps identify whether empowerment influences each service differently or consistently across the continuum of care, offering insights into potential gaps and reinforcing the need for integrated interventions.

Moreover, previous observational studies did not properly address confounding bias. To mitigate this, we aimed to apply propensity score matching (PSM) analysis to provide a more robust and efficient approach to account for confounding factors in non-experimental studies [16,17]. Additionally, while the association between women's empowerment and maternal healthcare use has been studied in individual countries, a multi-country approach using comparable Demographic and Health Survey (DHS) data offers broader insights that can be generalised across different LMIC settings.

We aimed to examine the impact of women’s empowerment on the uptake of maternal healthcare services (ANC visits, skilled birth attendance, and PNC visits) in LMICs using DHS data from 35 countries. For this study, we defined empowered women as currently married women who participate in all decision-making and who disagree with all of the reasons justifying wife-beating, which aligns with the DHS, our data source for analysis. We intended to provide stronger empirical evidence on the role of women’s empowerment in maternal healthcare uptake in order to guide policymakers in designing more targeted, effective, and equitable health interventions that are both country-specific and aligned with global maternal health goals.

## METHODS

### Study design and data source

We used the most recent cross-sectional DHS data from 35 LMICs in the era of Sustainable Development Goal programmes (post-2015) [18]. The DHS reports nationally representative cross-sectional data, which measures key policy-relevant and pragmatic indicators across LMICs. Its data is collected using standardised questionnaires, coding, data collection procedures, and sampling techniques, providing robust and comparable healthcare results across LMICs [19].

### Study population and eligibility

Out of 84 LMICs that had publicly available DHS data, we excluded those without data post-2015 (n = 46) or those that did not contain our exposure variable (n = 3), leaving the latest data from 35 countries for analysis (**Table 1**; Figure S1 in the **Online Supplementary Document**).

**Table 1:** Geographic, survey, and population characteristics of reproductive-age women from 35 LMIC countries (DHS 2016–23)

Countries by region	Survey year		Sample
**Sub-Saharan Africa**			
East Africa			
*Burundi*	2016/17		2454
*Ethiopia*	2019		2086
*Madagascar*	2021		2079
*Rwanda*	2019/20		1316
*Kenya*	2022		3342
*Uganda*	2016		2729
*Tanzania*	2022		1893
Southern Africa			
*Zambia*	2018		1508
*South Africa*	2016		248
Central Africa			
*Cameroon*	2018		1577
*Gabon*	2019/20		815
West Africa			
*Benin*	2017/18		2807
*Burkina Faso*	2021		2331
*Cote d’Ivoire*	2021		1897
*Gambia*	2019/20		1774
*Ghana*	2022		1664
*Guinea*	2018		1556
*Nigeria*	2018		6334
*Liberia*	2019/20		815
*Mali*	2018		1923
*Mauritania*	2020/21		2241
*Senegal*	2019		1245
*Sierra Leone*	2019		1718
**North Asia/Europe**			
Africa/West			
*Albania*		2017/18	532
Central Asia			
*Tajikistan*		2017	1129
South and Southeast Asia			
*Bangladesh*		2017/18	1804
*Cambodia*		2021/22	1692
*India*		2019/21	6951
*Indonesia*		2017	3369
*Temor-Leste*		2016	1483
*Nepal*		2022	1013
*Pakistan*		2017/2018	2523
*Philippines*		2022	1457
Oceania			
*Papua New Guinea*		2016/18	1692
Latin America and Caribbean			
*Haiti*		2016/17	1133

Our analysis focussed on women of reproductive age (15–49 years) who were married/partnered and had given birth in the year preceding the survey. Accordingly, of all women of reproductive age from LMICs during the survey period (n = 1 336 716), we considered 112 860 women who were married/partnered and had given birth in the year immediately before the survey. For ethical and safety considerations, the DHS used only one woman per household, randomly selected from all eligible women in the household for participation in the individual questionnaire of the spouse violence and women's empowerment module. Consequently, the number of women with data on women's empowerment will always be lower than the number of women selected for the full DHS individual interview [19,20]. Based on this, after excluding samples that did not report data on women empowerment (n = 41 783), we retained 71 077 samples reported both women empowerment and maternal health services uptake for the final analysis. Sample weighting was applied during the analysis to account for the differences in population sizes between clusters (Figure S2 in the **Online Supplementary Document**).

### Study variables

The outcome variables of interest was the uptake of maternal health services (having adequate ANC visits, health facility childbirth, and early PNC visits). Based on the WHO recommendation, we considered women to have adequate ANC visits if they had eight or more visits during their pregnancy period [21]. Moreover, we considered an early PNC visit to have happened if a woman has received at least one health checkup by a health professional within 2 days of childbirth [21] (Table S1 in the **Online Supplementary Document**).

The study's treatment (*i.e.* exposure) variable was women's empowerment. Given the wide variation in the definition of women's empowerment [22], it is challenging to measure consistently. To address this, the DHS Program has developed standardised questionnaires to assess women's empowerment, providing consistent and comparable measures across a wide range of countries [19]. For this study, since we used DHS data from LMICs, we adopted the DHS definition of women's empowerment to ensure consistency and comparability across countries. Consequently, the DHS defines empowered women as married or partnered women who participate in all decision-making items and disagree with all justifications for wife-beating [19]. Participation in decision-making is operationalised as currently married women usually making all three specific decisions either alone or jointly with their husband: on their own healthcare, on large household purchases, and regarding visits to family or relatives. Similarly, attitudes toward wife-beating are operationalised as women who disagreed that a husband is justified in hitting or beating his wife for all the reasons: going out without telling him; neglecting the children; arguing with him; refusing to have sexual intercourse with him; burning food (Table S2 in the **Online Supplementary Document**).

Based on the literature and the availability of information in the data set, we selected covariates associated with the treatment variables. Then, we assessed the difference in baseline covariates between the treatment and control groups using a chi-squared test. We selected significant covariates and further evaluated for their confounding effect on three outcome variables. Covariates that were associated with the treatment variable (women’s empowerment) and at least one of the three outcome variables were selected for the matching analysis. Based on this, we used covariates such as women's age, household head gender, women's education level, household wealth status, access to media, residence, geographical region, and country income status for matching in this analysis (Table S1 in the **Online Supplementary Document**).

### Data management and analysis

#### Choosing matching or weighting methods

We used PSM analysis to estimate the treatment effects by balancing the inequality of confounding variables. To generate the propensity score, we used women’s empowerment (treatment variable) as an outcome variable and confounders or matching variables as covariates using logistic regression analysis. Then, we compared and selected the types of matching or weighting methods by matching the treatment variable with one of the outcomes (having eight or more ANC visits) (Table S3 in the **Online Supplementary Document**). Based on the likelihood ratio test *P*-value and the level of mean or median bias, we selected the nearest neighbour matching approach with replacement as the best matching method. With a large sample size and women from similar backgrounds (LMICs), this matching approach proved to be more efficient. The likelihood ratio *P*-value (0.996) from this matching approach suggested that there was no statistically significant difference between the treatment and control groups after matching, indicating that the matching process better balances the covariates between the treated and control groups than other approaches. As a result, we matched a treated individual to the comparison individual with the most similar propensity score [23]. The mean propensity score was 0.31, with minimal variability (SD = 0.12) between the empowered and not empowered groups. There was no off-support in either group, or the region of common support ranged from 0.105 to 0.677 of the propensity score (Figure S3 in the **Online Supplementary Document**). Therefore, we used this matching approach to estimate the average treatment effect of having adequate ANC visits, health facility childbirth, and early PNC visits among the entire population (average treatment effect), in the treatment group or empowered women (average treatment effect on the treated), and the untreated or controls (average treatment effect on the untreated)).

#### Post-matching tests

We then conducted post-matching tests such as balancing and bias estimation analysis to evaluate the quality of the matching process in reducing differences between the treatment and control groups. We used a kernel density plot to evaluate the balance between the treatment and control groups (the overlap of propensity scores). Moreover, we assessed balancing tests using standardised differences and variance ratio tests to assess the level of balancing objectively. A covariate is considered perfectly balanced if it has a standardised difference of zero and a variance ratio of one [17,24]. Furthermore, we assessed the percentage of bias and bias reduction both graphically and statistically. For each covariate included in the propensity score estimation model, we reported percentage bias and percentages bias reduction along, with corresponding *P*-values.

### Handling missing data

The DHS guidelines specify the methods for handling missing data when defining the women's empowerment index. Missing values for who usually makes specific decisions are assumed to indicate someone other than the respondent. Similarly, missing values for attitudes toward wife beating are assumed to reflect disagreement or the belief that wife beating is not justified [19]. Moreover, we conducted our PSM analysis for complete cases of the outcome variables. Studies have shown that in cross-sectional studies with large data sets, when data are missing completely at random, complete case analysis is the preferred method for managing missing data [25].

## RESULTS

**Figure 1:**
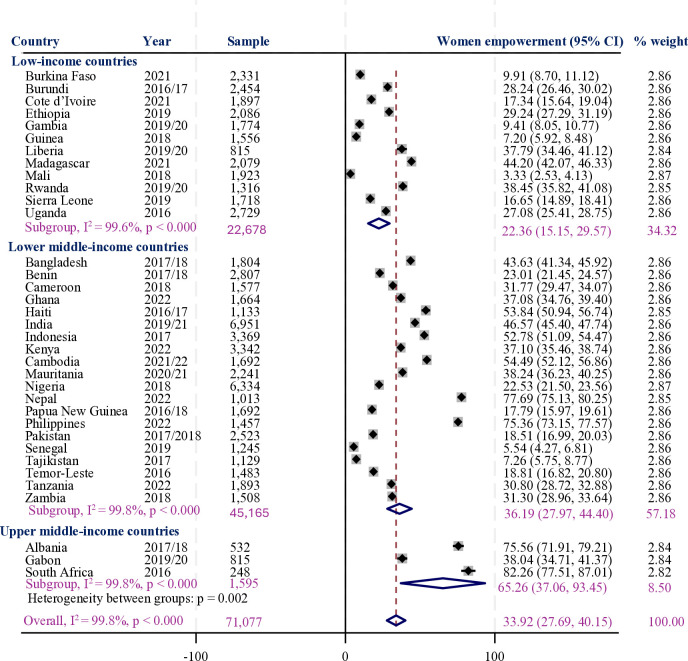
The proportion of women's empowerment among LMICs.

In our data, 33.9% (95% confidence interval (CI) = 27.7–40.8) of reproductive-aged women in LMICs were reported to be empowered. The proportion of empowerment ranged from the lowest figure in Mali (3.3% (95% CI = 2.5–4.1)) to the highest in South Africa (82.3% (95% CI = 77.5–87.1)). Notably, secondary and above-educated women (42%) and women from the highest-wealth households (38%) had higher rates of women’s empowerment compared to their non-educated counterparts (17%) and those from the lowest-wealth households (27%), respectively. Moreover, women from upper-middle-income countries were more likely to be empowered (65%) than those from lower-income countries (22%) (**Figure 1**, **Table 2**).

**Table 2:** The association between covariates with exposure and outcome variables

	Exposure variables		Outcome variables					
**Covariates**	**Women empowerment**	***P*-value**	Adequate ANC visit*	***P*-value**	**Health facility childbirth**	***P*-value**	**Early PNC visit**	***P*-value**
**Sample, n (%)**	21 905 (33.9)		8653 (11.7)		51 667 (74.9)		46 548 (67.2)	
**Maternal age**		<0.001		<0.001		<0.001		<0.001
15**–**24	6722 (26.9)		2618 (10.5)		18 336 (73.3)		16 462 (65.8)	
25**–**34	11 224 (33.1)		4694 (13.9)		24 852 (73.3)		22 487 (66.4)	
35**–**49	3959 (32.5)		1341 (11.0)		8.479 (69.7)		7599 (62.5)	
**Educational level**		<0.001		<0.001		<0.001		<0.001
Not educated	4045 (17.2)		1123 (4.8)		13 751 (58.4)		12 480 (52.9)	
Primary	5750 (30.6)		1455 (7.8)		13 306 (70.8)		11 543 (61.5)	
Secondary and above	12 110 (42.2)		6075 (21.1)		24 610 (85.7)		22 525 (78.4)	
**Household head gender**		<0.001		0.05		<0.001		<0.001
Male	18 044 (29.9)		7282 (12.1)		43 340 (71.9)		39 216 (65.1)	
Female	3861 (35.8)		1371 (12.7)		8327 (77.1)		7332 (67.9)	
**Household wealth status**		<0.001		<0.001		<0.001		<0.001
Low	8945 (26.5)		2617 (7.8)		20 403 (60.5)		19 372 (57.4)	
Middle	4041 (29.0)		1687 (12.1)		10 545 (75.7)		9290 (66.7)	
High	8919 (38.1)		4349 (18.6)		20 719 (88.6)		17 886 (76.4)	
**Household-level media exposure**		<0.001		<0.001		<0.001		<0.001
No	5967 (25.3)		1375 (5.8)		13 797 (58.5)		12 363 (52.4)	
Yes	15 926 (33.6)		7273 (15.3)		37 851 (79.8)		34 165 (72.0)	
**Country income status**		<0.001		<0.001		<0.001		<0.001
Low	4964 (21.9)		1048 (4.6)		16 434 (72.5)		14 227 (62.7)	
Lower-middle	16 025 (34.2)		7320 (15.6)		33 751 (72.1)		31 063 (66.4)	
Upper-middle	916 (62.5)		285 (17.9)		1482 (93.0)		1258 (78.9)	
**Residence**		<0.001		<0.001		<0.001		<0.001
Urban	8330 (37.3)		4238 (19.0)		19 310 (86.5)		16 926 (75.8)	
Rural	13 575 (27.9)		4415 (9.1)		32 357 (66.4)		29 622 (60.8)	
**Region**		<0.001		<0.001		<0.001		<0.001
Sub-Saharan Africa	11 890 (25.7)		3387 (7.3)		32 883 (70.9)		28 368 (61.2)	
North Africa/West	402 (75.6)		164 (30.8)		530 (99.6)		472 (88.7)	
Asia/Europe Central Asia	82 (7.3)		182 (16.1)		1014 (89.8)		1037 (91.9)	
South and Southeast Asia	8620 (42.5)		4619 (22.8)		15 834 (78.1)		15 428 (76.1)	
Oceania	301 (18.3)		156 (9.5)		995 (60.4)		871 (52.8)	
Latin America and Caribbean	610 (53.8)		145 (12.8)		411 (36.3)		372 (32.8)	

ANC – antenatal care visits, PNC – postnatal care

*Adequate ANC was defined when women reported recipient of at least eight ANC visits.

### Maternal healthcare utilisation in LMICs

In our sample, only 11.6% (95% CI = 9.7–13.6) of women reported having an adequate number of ANC visits. The highest adequate ANC uptake was observed in Indonesia (54.1%), while the lowest was observed in Senegal (0.08%). Additionally, women from upper-middle-income countries had more adequate ANC visits (20.5%) than those from lower-income countries (5.35%) (Figure S4 in the **Online Supplementary Document**).

**Figure 2:**
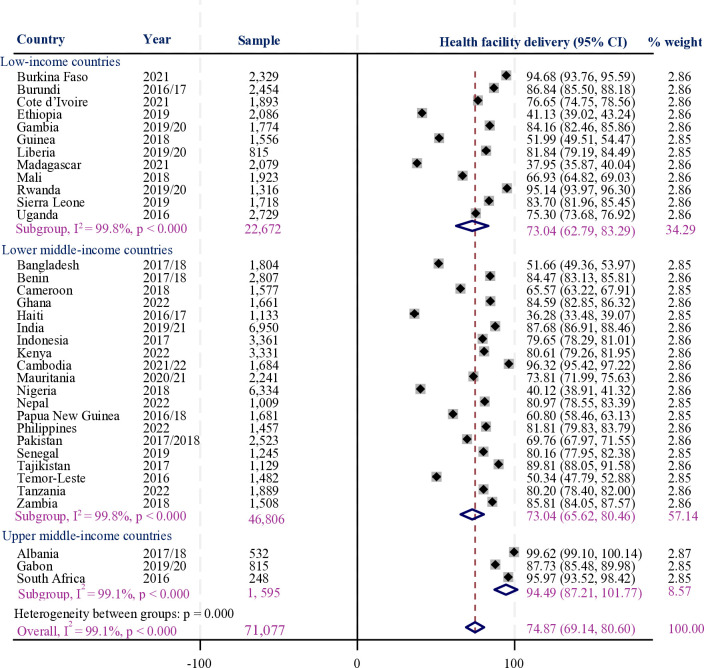
The proportion of women who had a health facility childbirth in LMICs.

Moreover, 74.8% (95% CI = 69.1–80.6) of women gave birth in health facilities, with the highest proportion observed in Albania (99.6%) and the lowest in Haiti (36.7%). Most women from upper-middle-income countries (94.5%) had a health facility childbirth, compared to less than three-fourths (73%) of women from low-income or lower-middle-income countries (**Figure 2**).

Lastly, 67.2% (95% CI = 60.8–73.5) reported early PNC visits, with the lowest uptake observed in Ethiopia (21.2%) and the highest in Bangladesh (90.8%). Moreover, women from upper-middle-income countries had more PNC visits (81.8%) than those from lower-income countries (64.4%) (Figure S5 in the **Online Supplementary Document**).

### The association between women empowerment and maternal health services

We found the ATT for having adequate ANC visits in the treatment group and the control group were 0.17 and 0.06, respectively, with an ATT difference of 0.11 (standard error (SE) = 0.026). This means that if women were empowered, an additional 11% of women would achieve adequate ANC visits. Similarly, the ATT of health facility childbirth was eight percentage points (ATT = 0.078, SE = 0.039) greater for the empowered group compared to the unempowered group (*β* = 0.71). However, there was a non-significant decrease in ATT of having early PNC visits on empowered (*β* = 0.72) as compared to unempowered groups (*β* = 0.76), with an ATT difference of −0.04 (SE = −0.043) (**Table 3**).

**Table 3:** Unmatched and matched estimates of women's empowerment on maternal healthcare utilisation

Variable	Sample	Treated	Control	Difference	SE	*P*-value	T-stat
Adequate ANC visit	Unmatched	0.171	0.099	0.071	0.003	<0.001	27.00
	ATT	0.171	0.059	0.112	0.026	<0.001	4.34
	ATU	0.099	0.041	−0.058			
	ATE			−0.006			
Health facility childbirth	Unmatched	0.791	0.698	0.093	0.004	<0.001	25.80
	ATT	0.791	0.713	0.078	0.039	0.042	1.99
	ATU	0.698	0.74	0.085			
	ATE			0.08			
Early PNC	Unmatched	0.719	0.636	0.093	0.004	<0.001	24.15
	ATT	0.719	0.762	−0.043	0.041	0.053	−1.97
	ATU	0.636	0.759	0.133			
	ATE			0.067		<0.001	

ATT – average treatment on treated, ATU – average treatment on untreated, ATE – average treatment effect, ANC – antenatal care, PNC – postnatal care, SE – standard error

### Post matching tests

#### Balancing of propensity score across empowered and unempowered groups

Studies have shown no specific rule regarding the acceptable level of imbalance in a propensity score. However, a maximum standardised difference for specific covariates is less than 10%, and a variance ratio near one is considered acceptable [23]. In our analysis, the matched standardised differences for all covariates were close to zero (*i.e.*<10%) and the variance ratios for all covariates were close to one compared to the unmatched cohort. This showed that the propensity score balance was improved between the two groups of women when the data were matched (Table S4 in the **Online Supplementary Document**). Moreover, the density plot results indicate that the distributions of the propensity scores perfectly overlapped after matching, which achieved a balance of the propensity score distribution between exposed and non-exposed women (Figure S6 in the **Online Supplementary Document**).

#### Bias estimation analysis

**Figure 3:**
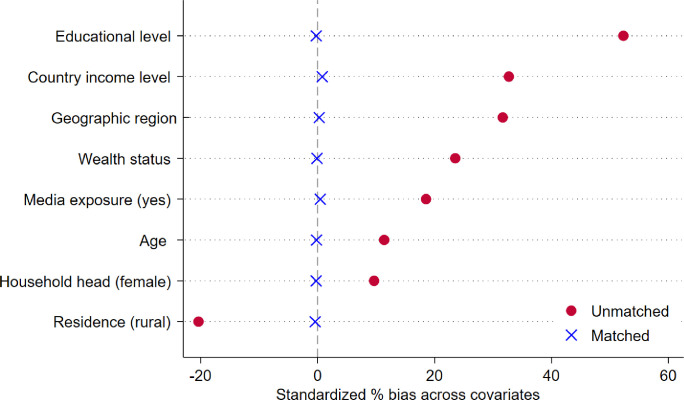
Standardised percentage of bias before and after matching across covariates.

In our analysis, we observed that all covariates were sufficiently balanced after matching, and the percentage of bias reduction ranged from household head gender (97.2%) to women's education status (99.5%). The low value of pseudo-R^2^ (0.001) indicates that the distribution of covariates between both groups was similar after matching (Table S5 in the **Online Supplementary Document**). After conducting PSM analysis, the standardised percentage bias across all covariates is almost close to zero (**Figure 3**).

## DISCUSSION

Here we analysed analysed multi-country nationally representative data to examine the association between women's empowerment and maternal health services uptake in LMICs using PSM analysis. This approach is useful for assessing the effect of a single exposure variable on multiple outcomes, as done in this study, making it easier to compare them. Our use of PSM aimed to generate high-quality evidence equivalent to randomised controlled trials [26,27] to provide insights into treatment effects, policy implications, practice, and programme evaluations related to maternal healthcare use.

Based on this, we found that only about one-third of women in LMICs were reported to be empowered. We also found that, unlike early PNC uptake, women's empowerment was associated with increased uptake of adequate ANC and childbirth in health facilities.

In our sample, only about one-third of reproductive-age women in LMICs were empowered. Similarly, the World Economic Forum Global Gender Gap Index 2023 report showed that only 68.4% of the gender gap was closed globally [28], though having different attributes with empowerment. This finding highlights the persistent structural, cultural, and socioeconomic barriers that hinder women's empowerment in these settings. Previous studies have shown that deeply entrenched patriarchal norms in many LMICs continue to restrict women’s autonomy, limiting their participation in household decision-making and reinforcing the acceptance of gender-based violence [29]. Societal attitudes that normalise wife-beating and prioritise male authority over household decisions further constrain women’s ability to exercise agency over their own lives [30]. Moreover, economic dependence is another critical barrier to women's empowerment. Many women in LMICs lack access to financial resources, formal employment, and economic opportunities, which can reduce their bargaining power within households [31]. Limited educational attainment exacerbates this challenge, as education is a key determinant of women's ability to engage in decision-making and resist harmful societal norms [32]. Additionally, the inadequate enforcement of gender-equality policies, including those aimed at addressing domestic violence and promoting women's rights, weakens the impact of legal frameworks intended to safeguard and empower women [33].

There is a wide range of differences in the proportion of empowerment among LMICs, ranging from the lowest in Mali (3.3%) to the highest in South Africa (82.3%). Similarly, reports showed that South Africa is the highest in sub-Saharan Africa (SSA), having closed 70% of the overall gender gap, whereas Mali is among the lowest-performing countries, with scores below 62% [28]. A multidimensional women's empowerment study in Mali also reported a relatively low level of women's empowerment [34]. The differences in women's empowerment levels across LMICs, such as the higher empowerment observed in South Africa compared to the lower levels in Mali, can be attributed to a combination of attributes such as economic status, policy frameworks, and sociocultural norms. Mali is a low-income country, while South Africa is the wealthiest country in SSA, with upper-middle-income status [35]. Studies showed that an increase in family wealth [36] and the implementation of economic development programmes [37,38] improve the level of empowerment of women. In South Africa, social protection programmes such as child support grants also contribute to women’s financial independence [39] unlike in Mali, economic dependence on males.

Moreover, differences in policies and legal frameworks play a pivotal role in this significant disparity. For example, South Africa has progressive gender policies and legal protections that promote women's rights, including the Promotion of Equality and Prevention of Unfair Discrimination Act 4[40] and the Domestic Violence Act [41]. These policies enhance women's autonomy by ensuring legal protection against gender-based violence and discrimination including women beating. In contrast, Mali has weaker enforcement of gender equality laws, with customary and religious laws often taking precedence over statutory laws [42] restricting the practice of women's rights.

Education and sociocultural norms also play a crucial role in shaping women's autonomy and decision-making. Some LMICs, such as Mali, have deeply entrenched patriarchal norms that restrict women’s decision-making ability, with early marriage and lower educational attainment rates among women serving as barriers to empowerment. For example, female literacy rates exceed 90% in South Africa [43], while only about 30% of adult women in Mali were literate [44]. Addressing this disparity requires context-specific intervention that integrates the policy reforms, economic inclusion of women, improving educational opportunities, and promoting gender-balanced household dynamics [34].

In our study, empowering women could increase the likelihood of approximately 11 percentage points (SE = 0.026) of women attending adequate ANC visits. This finding is supported by studies in SSA [45], Southeast Asian countries [14], and developing countries [46] which showed that women's empowerment had higher odds of attending ANC visits. This finding parallels the concepts observed in our study, which is that empowered women may produce normative changes in gender relations and women’s family and community roles. With higher levels of empowerment, women may improve their status at the domestic level, maintain their health and seek necessary health-related resources. However, a pooled analysis of DHS data from SSA showed that women's empowerment did not predict the use of skilled ANC visits [47]. One difference between this study and ours is that our response variable is eight or more ANC visits, whereas the second study focussed on any skilled ANC service for the most recent birth.

Our analysis indicates that women's empowerment also increases the likelihood of childbirth in health facilities by eight percentage points. This finding is supported by a multi-country analysis in SSA [15] and a study in developing countries [46] which showed that women with the highest empowerment scores were more likely to have a skilled attendant at birth compared to those with no empowerment scores. This is because decision-making power regarding their healthcare is one of the elements of women's empowerment. This autonomy allows them to make informed choices about delivering in health facilities, which eventually leads to improved maternal and neonatal outcomes [48].

Our research showed that women's empowerment did not have a significant association with early PNC service utilisation. This is different from other studies [49,50], which showed the positive odds of women's empowerment on early PNC visits. One possible explanation for the lack of a significant association between women's empowerment and early PNC service utilisation in our study is the mode of PNC service delivery. In many LMICs, PNC services are not necessarily facility-based. Instead, they are often provided through home visits by health extension workers or community health workers [51]. Unlike ANC or facility-based delivery, which require women to actively seek care at a health facility, PNC services can be delivered directly to their homes [19]. In most developing countries, PNC may only be received through home visits, as geographic, financial, and cultural barriers often limit access to care outside the home during the early postnatal period [51]. Empowerment is mainly defined in terms of decision-making power and the rejection of wife-beating, in this study. However, PNC largely depends on the performance of the health system, particularly outreach services (*e.g.* home visits). This means that even women with lower levels of empowerment may still receive early PNC if the health system is effective, as they do not need to make independent healthcare decisions. This may also relate to cultural and societal factors. In some settings, cultural beliefs may not emphasise early PNC as much as pregnancy care and delivery [52]. Women may be empowered; however, they might perceive PNC as unnecessary if they have a safe delivery without complications. Consequently, there may be no significant difference in service uptake between empowered and unempowered women. This suggests that women’s empowerment alone may not be enough to improve early PNC utilisation if services are primarily delivered through outreach. Policies should also ensure that health system performance is robust enough to reach women at home, especially in rural and underserved areas.

This study has several strengths. First, we used the PSM analysis, which is the most used approach for controlling selection bias in observational studies when randomisation is not feasible [16,17], to provide insights into treatment effects, policy implications, practice, and programme evaluations related to maternal healthcare. This approach is also useful for assessing the impact of a single exposure variable on multiple outcomes, making it easier to compare, as we have done in this study. Further, PSM is preferred over other effect analysis methods, such as instrumental variable analysis, as it reduces selection bias while maintaining a large sample size. Moreover, to minimise recall bias, we focussed on women who had live births in the year preceding the surveys.

However, it is important to note some limitations of our study. First, PSM matches the treated with controls based on measured covariates, and as a result, bias due to unmeasured covariates might occur. Therefore, unmeasured variables such as cultural norms, policy interventions, and differences in health system capacity across countries should be considered when interpreting these results. Second, by including only women who had live births, there is a possibility of selection bias, even though a recent methodological review suggests that the bias resulting from the restriction to the analysis of live births may be minimal [53]. The other limitation of this study is that, since we included only countries with DHS data available after 2015, less than half of the LMICs with publicly available DHS data were included. Most of the countries were excluded due to a lack of recent DHS data, while others were missing exposure variables. This may introduce selection bias if the excluded countries or women differ systematically from those included in terms of maternal healthcare policies, health system capacity, or socioeconomic factors.

Moreover, although we defined adequate ANC visits as a minimum of eight, aligning with WHO guidelines that emphasise comprehensive maternal care, it is important to acknowledge that, in some LMICs, resource constraints often limit ANC access, making the four-visit model more prevalent in these regions. This may impact the generalisability of our findings. In addition, while women's empowerment is a multidimensional concept with varying definitions, our study adopts the DHS women's empowerment indicator, which primarily focusses on autonomy in decision-making and the rejection of wife-beating. This indicator encompasses certain human rights, such as freedom of movement, access to healthcare, financial management, and protection from violence, including physical and sexual violence. However, this measure may not fully capture the broader dimensions of women's empowerment, such as educational, economic, and social aspects. Future research could explore additional indicators to provide a more comprehensive understanding of women's empowerment.

## CONCLUSIONS

Only about one-third of women in the sampled LMICs were estimated to be empowered. Women’s empowerment significantly increased the recommended ANC visits and health facility childbirth in LMICs, underscoring a critical opportunity for transformative policy and programmatic action. These results highlight that women’s empowerment is not merely a social justice issue but a cornerstone of effective public health strategies. Empowerment initiatives that enhance women's decision-making power and autonomy can serve as pivotal mechanisms to improve maternal health outcomes. By prioritising these strategies, policymakers can increase ANC utilisation and health facility childbirth, ultimately reducing maternal mortality and morbidity in LMICs. Furthermore, public health programmes should consider incorporating empowerment interventions into existing maternal health services. This integration can be achieved through community-based approaches that promote women's education, economic empowerment, and social support networks.

Further research is needed to explore the specific pathways through which women's empowerment influences maternal health outcomes. Qualitative studies could explore the lived experiences of empowered women, elucidating the barriers they face and the facilitators that enable them to access and utilise maternal health services effectively. Additionally, longitudinal studies could assess the long-term impact of empowerment interventions on maternal health service uptake, providing robust evidence for scalability and sustainability.

## Additional material


Online Supplementary Document
