# Supplementary material for: The role of women's empowerment in the uptake of maternal health services in low- and middle-income countries: a propensity score-matched analysis
Source: J Glob Health. 2025 Jun 20;15:04188. doi: 10.7189/jogh.15.04188 (PMC12180484; doi:10.7189/jogh.15.04188)
Supplement: Online Supplementary Document [file jogh-15-04188-s001.pdf]

Supplement to: Belay DG, Tessema GA, Dunne J, Roy A, Norman R. The role of women's empowerment in the uptake of maternal health services in LMICs: a propensity score-matched analysis. J Glob Health. 2025;15:04188.

Table S1. Study variables and measurement of the effect of women's empowerment on maternal healthcare uptake in LMIC (DHS 2016-2022)

Level	Variables	Measurements
Outcome variables	ANC visit	If a woman had at least eight ANC visits (measured as yes/no) throughout their pregnancy.
	Health facility childbirth	Includes delivery that occurred in public, private, or non-governmental organisation (NGO) affiliated health facilities for the last birth.
	Early PNC visit	If a woman had received at least one health checkup by a health professional within 2 days of birth.
Treatment variables	Treatment group (women empowerment)	Married/partnered women who were categorised as *empowered* based on their report in participating in all decision-making processes and disagree with all reasons justifying wife-beating unless they are not empowered [19].
	Control group (not having women's empowerment)	Married women who were not categorised as empowered based on the DHS under the Women Empowerment Index [19].
	Age	The age of women was categorised as 15-19, 20-35, and 36-49.
	Education level	The highest educational attainment of the women and categorised as not educated, primary, secondary, and above educational status.
	Household head gender	Male or female

Covariates	Wealth index	The datasets contained a wealth index that was created using principal components analysis coded as lowest, low, middle, high, and highest (poorest, poorer, middle, richer and richest) in the DHS data set respectively.
	Media Access	A composite variable was obtained by combining whether a respondent reads a newspaper/magazine, listens to the radio, and watches television with a value of "0" if women were not exposed to at least one of the three media, and "1" if a woman has access/exposure to at least one of the three media.
	Residency	Urban or rural, based on where the household lives.
	Country income status	The country's income status was categorized as a low-income, lower-middle-income, and upper-middle-income country based on the World Bank List of Economies classification since 2021[38]. World Bank calculated country income based on Gross National Income (GNI) per capita, which is categorised as low income $1,035 or less; lower middle income, $1,036-4,045, upper middle income $4,046-12,535, and high income $12,535 or more[27].
	Geographic region	1) Sub-Saharan Africa 2) North Africa/West Asia/Europe 3) Central Asia 4) South & Southeast Asia 5) Oceania 6) Latin America & Caribbean

Table S2. Treatment or exposure variables in the study of the effect of women's empowerment on maternal healthcare uptake in LMIC (DHS 2016-2022)

Variables	Measurements	Response
Attitudes toward wife-beating	In your opinion, is a husband justified in hitting or beating his wife in the following situations:<br>a) If she goes out without telling him?<br>b) If she neglects the children?<br>c) If she argues with him?<br>d) If she refuses to have sex with him?<br>e) If she burns the food?	 ☐YES ☐NO ☐Don't know<br>☐YES ☐NO ☐Don't know<br>☐YES ☐NO ☐Don't know<br>☐YES ☐NO ☐Don't know<br>☐YES ☐NO ☐Don't know
Participation in decision-making	Who usually makes decisions about:<br>a) Health care for yourself?<br>b) Making large household purchases?<br>c) Visits to your family or relatives?	☐Respondent alone ☐Husband/partner ☐Respondent and husband/partner jointly ☐Someone else ☐Other<br>☐Respondent alone ☐Husband/partner ☐Respondent and husband/partner jointly ☐Someone else ☐Other<br>☐Respondent alone ☐Husband/partner ☐Respondent and husband/partner jointly ☐Someone else ☐Other
Women empowerment	Married or partnered women who participate in all decision-making items (answered 'respondent alone' or 'jointly with their husband/partner') and disagree with all justifications for wife-beating (answered 'not justified').	

Table S3. Covariate bias checks for women's empowerment

Variable	Sample	Mean Treated	Mean Control	% Bias	% reduction bias	t-test	p>t
Age of women	Unmatched	0.87	0.79	11.4	98.1	13.9	<0.001
	Matched	0.87	0.87	-0.2		-0.2	0.82
Education status of women	Unmatched	1.37	0.94	52.3	99.5	63.2	<0.001
	Matched	1.37	1.37	-0.3		-0.3	0.78
Household head gender	Unmatched	0.18	0.14	9.7	97.2	12.1	<0.001
	Matched	0.18	0.18	-0.3		-0.3	0.78
Household wealth status	Unmatched	0.99	0.79	23.5		29.2	<0.001
	Matched	0.99	0.99	-0.1	99.5	-0.1	0.89
Media usage	Unmatched	0.73	0.64	18.6		22.5	<0.001
	Matched	0.73	0.72	0.4	97.7	0.5	0.64
Residence	Unmatched	1.62	1.71	-20.4		-25.4	<0.001
	Matched	1.62	1.62	-0.4	97.8	-0.4	0.66
Country income	Unmatched	0.82	0.65	32.7		39.9	<0.001
	Matched	0.82	0.81	0.8	97.7	0.8	0.41
Geographic region	Unmatched	2.40	1.92	31.7		39.7	<0.01
	Matched	2.40	2.39	0.2	99.2	0.25	0.80

Model significance	Sample	Ps R2	LR chi2	p-value	Mean bias	Median bias
	Unmatched	0.062	5476.5	<0.001	25.0	22.0
	Matched	0.001	1.72	0.988	0.3	0.3

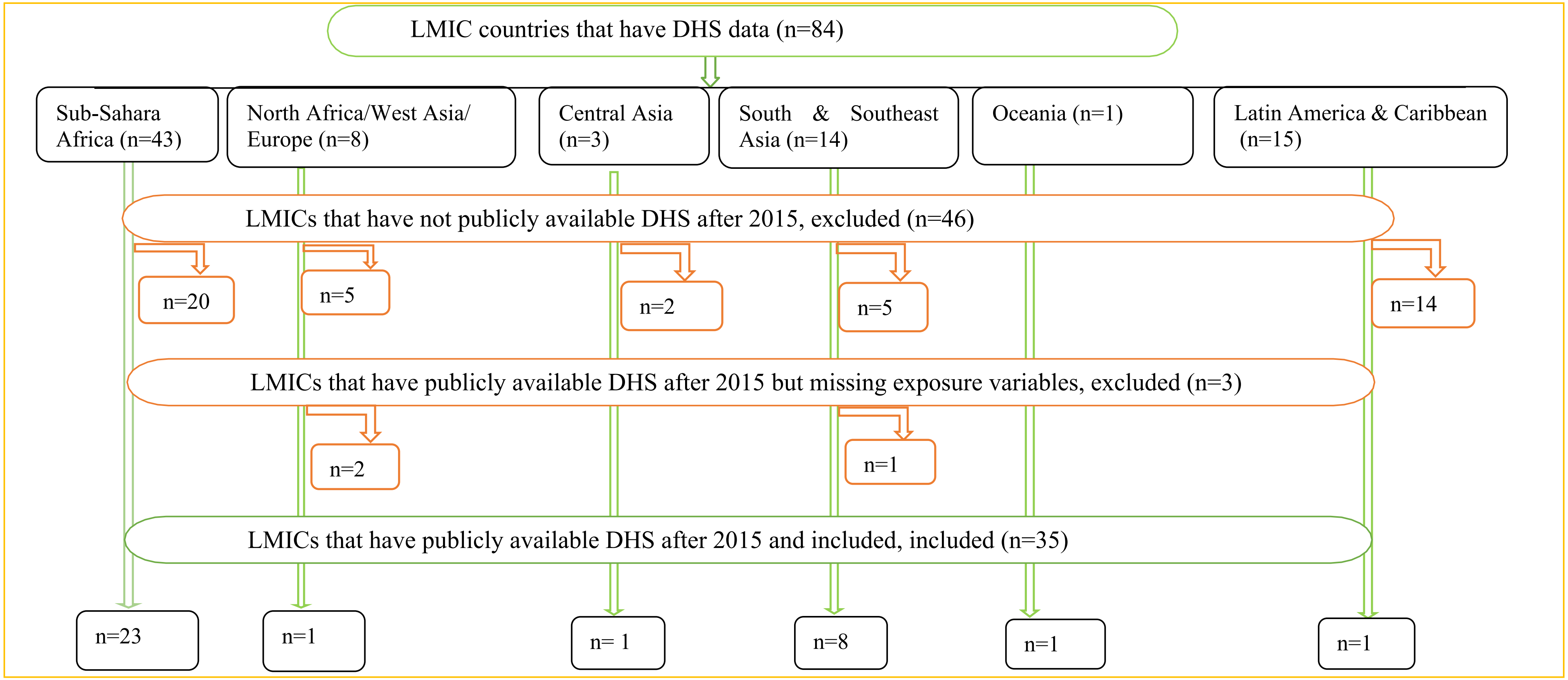

Figure S1. Schematic presentation of the sample selection.

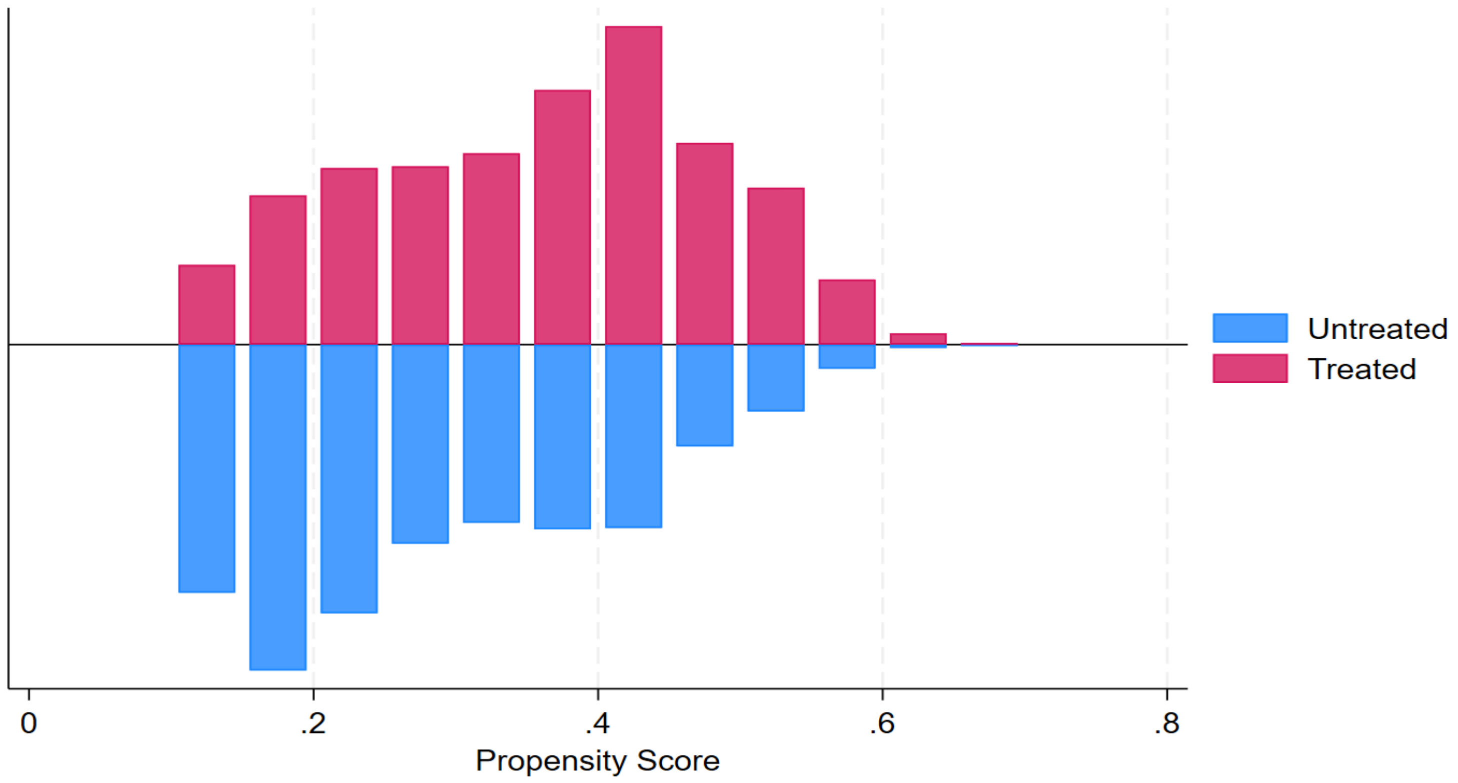

Figure S2. Propensity score histogram by treatment status (women empowerment).

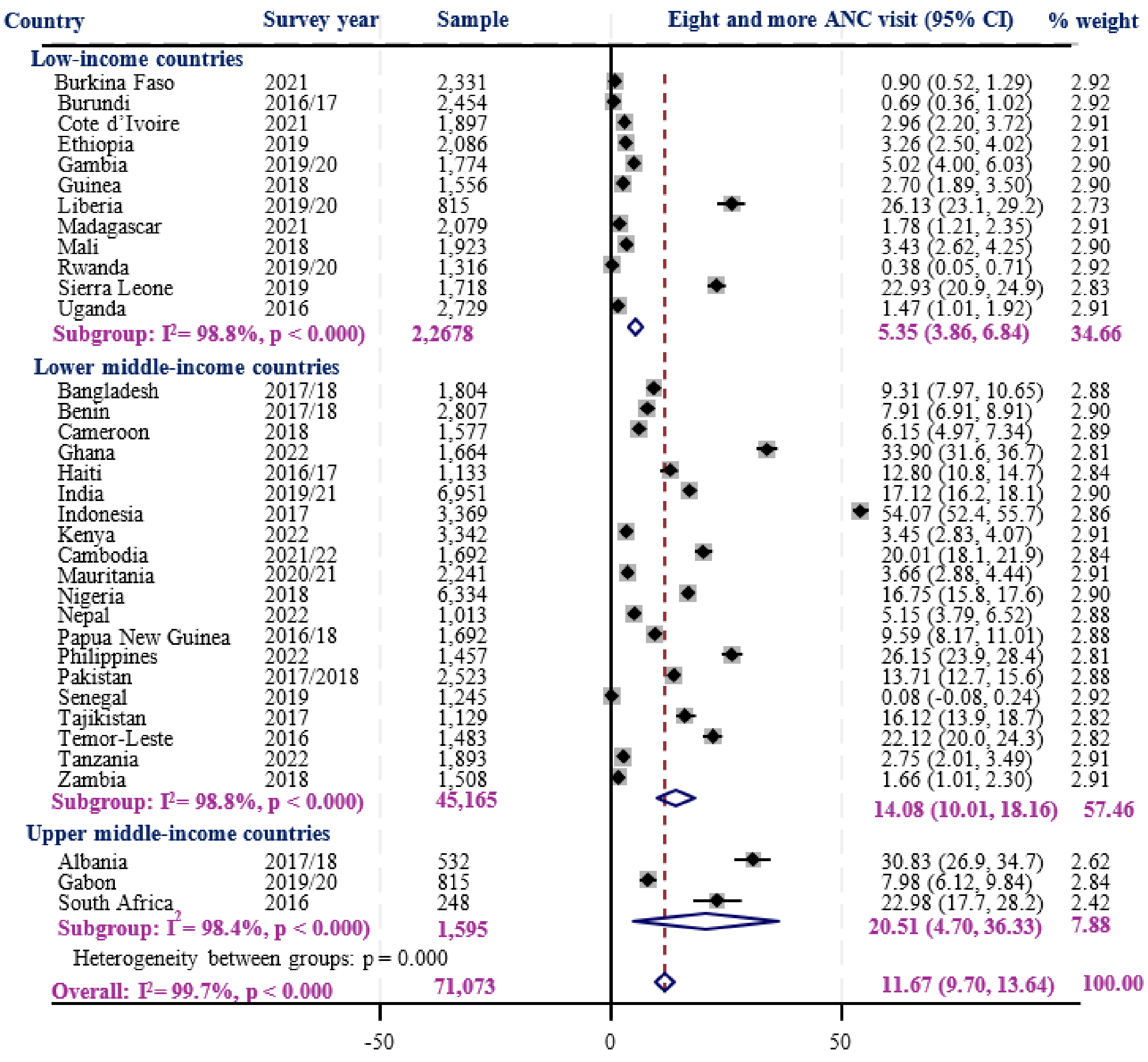

Figure S3. The proportion of women who took adequate ANC visits in LMICs.

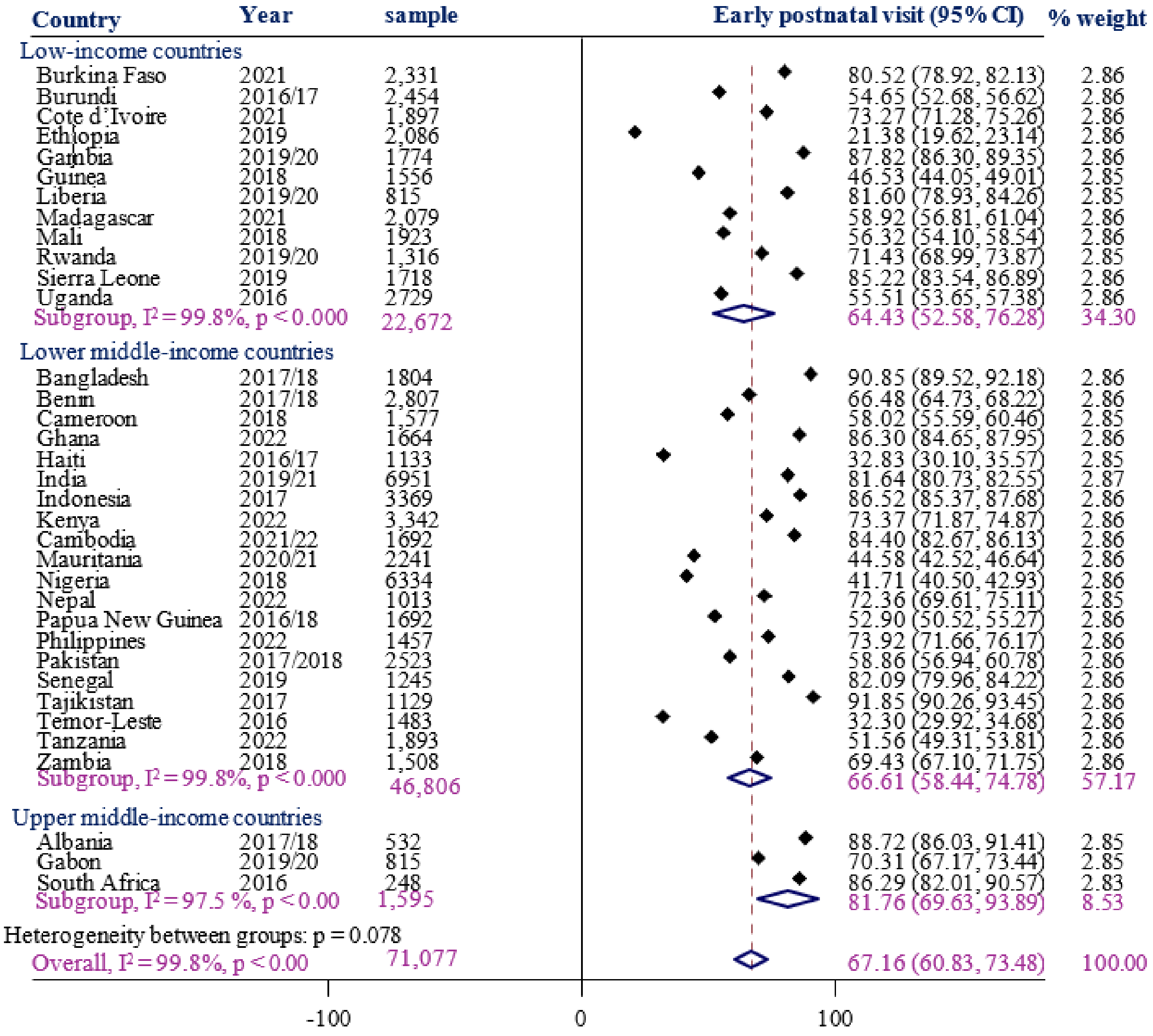

Figure S4. The proportion of women who took early PNC visits in LMICs.

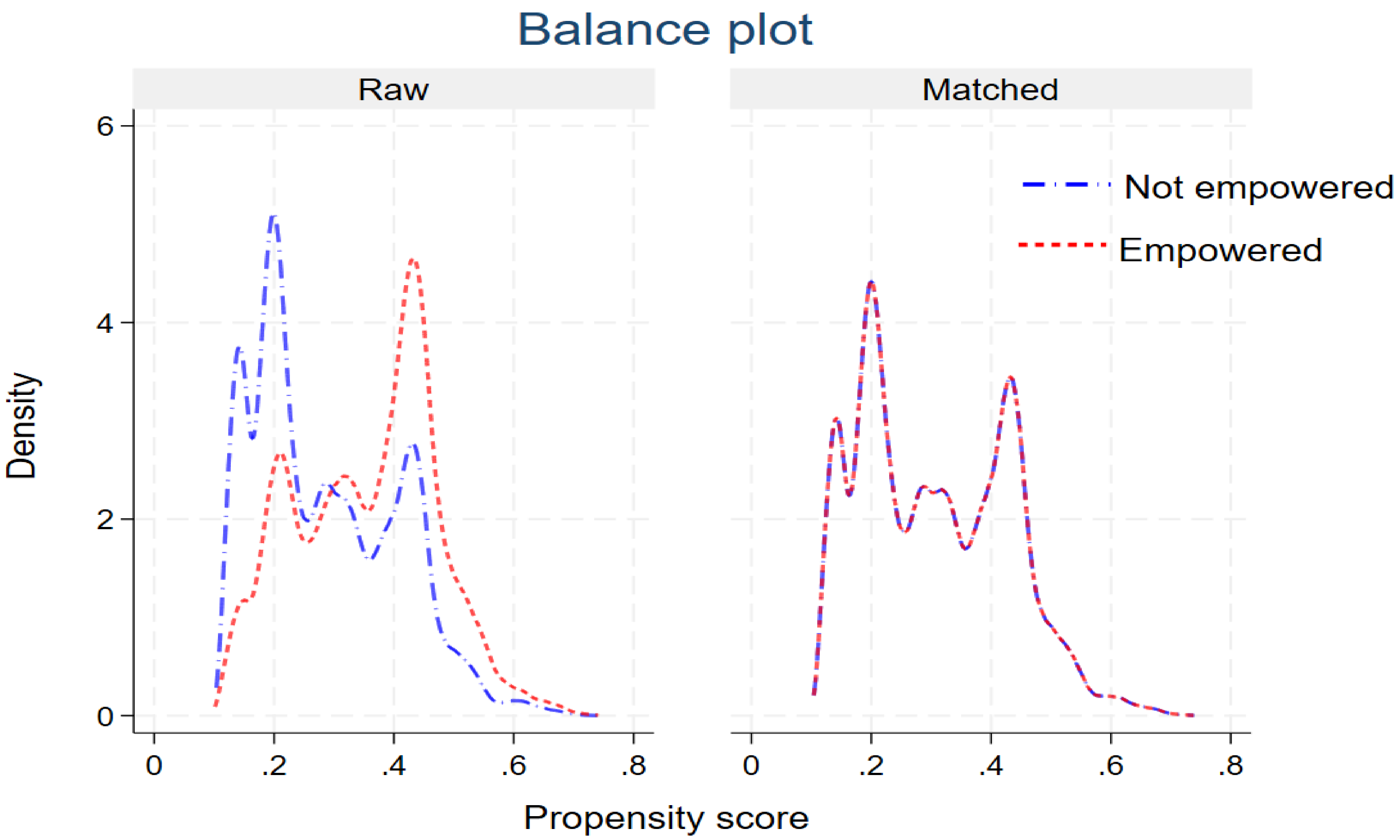

Figure S5. Kernel density plot of estimated propensity scores before and after matching for women's empowerment.
